# Clostridium septicum Bacteraemia Complicated by Spontaneous Gas-Forming Myositis Revealing a Suspected Caecal Cancer

**DOI:** 10.7759/cureus.117442

**Published:** 2026-10-04

**Authors:** Wajeeha Iqbal, Mohammed Mubin, Pasipanodya Guti, Payal Subhash Joshi

**Affiliations:** 1 General Medicine, Worcestershire Acute Hospitals NHS Trust, Redditch, GBR; 2 Geriatrics, Sandwell and West Birmingham NHS Trust, Birmingham, GBR; 3 Diabetes and Endocrinology, Worcestershire Acute Hospitals NHS Trust, Redditch, GBR

**Keywords:** bacteraemia, caecal carcinoma, clostridium septicum, frailty, gas-forming myositis, palliative care

## Abstract

*Clostridium septicum* is a Gram-positive, spore-forming anaerobic bacillus that is rarely isolated from blood cultures but is strongly and specifically associated with occult gastrointestinal malignancy, particularly colorectal carcinoma. We describe a 79-year-old woman with a Clinical Frailty Scale score of 6 who presented with vomiting, diarrhoea, fever, rigors, and breathlessness against a background of transitional cell carcinoma treated with nephroureterectomy, type 2 diabetes mellitus, a previous lacunar infarct, and severe ischaemic cardiomyopathy. Blood cultures grew Gram-positive rods identified as *Clostridium septicum*, and intravenous meropenem was started. Her admission was complicated by unexplained right leg swelling four days into admission and, the following day, right hip pain with reduced range of movement. Magnetic resonance imaging demonstrated a peri-articular collection with gas foci, and computed tomography revealed gas within the right obturator internus and piriformis muscles alongside an irregular, polypoidal caecal lesion suspicious for malignancy, provisionally staged on imaging as T2N0M0. The collection was too small to drain and was managed conservatively with a six-week course of antibiotics, with a sustained fall in inflammatory markers. Given her frailty and multiple comorbidities, the multidisciplinary team elected not to pursue biopsy and to proceed directly to palliative rather than curative management; she was discharged to a nursing home with community palliative follow-up. Clinicians who isolate *Clostridium septicum* from blood cultures should have a low threshold for musculoskeletal and colonic imaging, since frailty may preclude both histological confirmation and curative treatment even when the underlying association with malignancy is strongly suspected.

## Introduction

*Clostridium septicum* is a Gram-positive, anaerobic, spore-forming bacillus. Bacteraemia with this organism is uncommon, but when it does occur, mortality is high, and reported complications range from cerebral septic emboli (infected clots that travel to the brain) [1] to endophthalmitis (infection inside the eye) [2], spontaneous gas gangrene (a rapidly spreading, gas-producing infection of muscle) [3], and other unusual presentations. What sets *C. septicum* apart from other clostridia is its association with malignancy. Unlike *Clostridium perfringens*, which usually causes gas gangrene after trauma or surgery, *C. septicum *can produce spontaneous, non-traumatic myonecrosis (death of muscle tissue) with no obvious portal of entry, most often at the site of a colorectal tumour, and endoscopists have recommended examining the caecum whenever the organism is isolated from blood [4]. Most reported cases have been linked to colonic adenocarcinoma, particularly of the caecum [5], while central nervous system involvement, including pneumocephalus (air within the skull), has also been described [6]. A number of case reports have described *C. septicum* bacteraemia as the first indication of an otherwise undiagnosed colonic malignancy, well before the underlying cancer would have come to light through other means. In practice, this means a positive blood culture for *C. septicum *should trigger two separate lines of action: prompt, aggressive treatment of the sepsis and a search for an occult colorectal tumour, even if the patient's presenting complaint points elsewhere entirely [7]. We describe a case in which *C. septicum* bacteraemia, complicated by gas-forming myositis in the hip and pelvis, led to the discovery of a suspected caecal malignancy in a frail older woman.

## Case presentation

A 79-year-old woman with a Clinical Frailty Scale score of 6 [8] was admitted with a several-day history of vomiting, diarrhoea, fever, rigors, breathlessness, and reduced mobility. She had a background of transitional cell carcinoma of the renal pelvis (G2pTa), treated previously with nephroureterectomy, type 2 diabetes mellitus, a right lacunar infarct, and a silent myocardial infarction complicated by severe left ventricular dysfunction and cardiogenic shock (ejection fraction 11%). On examination, air entry was reduced at the left lung base, heart sounds were normal, and she had tenderness in the right abdomen. Blood tests on admission showed a white cell count of 15.3 × 10⁹/L with marked neutrophilia and lymphopenia, alongside a markedly elevated C-reactive protein (CRP) (267 mg/L) and a raised lactate (2.2 mmol/L), reflecting a significant systemic inflammatory response at presentation. Renal function showed hyponatraemia and an acute kidney injury (creatinine 169 µmol/L; estimated glomerular filtration rate (GFR) 25 mL/min). Liver function tests showed low total protein and hypoalbuminaemia, with a disproportionate rise in alanine aminotransferase (ALT) relative to alkaline phosphatase and a mildly elevated bilirubin, in keeping with a predominantly hepatocellular pattern of liver injury in the context of sepsis; potassium and globulin were within their reference ranges (Tables 1-3). Arterial blood gas analysis showed type 1 respiratory failure on supplemental oxygen with a metabolic alkalosis. Blood cultures grew Gram-positive rods, subsequently identified as *Clostridium septicum*, and she was started on intravenous antibiotics on microbiology advice. 

**Table 1:** Coagulation screen and full blood count with white cell differential on admission. PT: prothrombin time; INR: international normalised ratio; APTT: activated partial thromboplastin time; Hb: haemoglobin; WBC: white blood cell count; PLT: platelet count; RBC: red blood cell count; HCT: haematocrit; MCV: mean cell volume; MCH: mean cell haemoglobin; MCHC: mean cell haemoglobin concentration; L: low; H: high.

Test	Result	Unit	Reference range	Flag
Coagulation screen				
Prothrombin time (PT)	14	s	9–14	
PT ratio (INR)	1.2	–	0.8–1.2	
Activated partial thromboplastin time (APTT)	25	s	24–35	
APTT ratio	0.8	–	0.8–1.2	
Full blood count				
Haemoglobin (Hb)	107	g/L	115–164	L
White cell count (WBC)	15.3	×10^9^/L	4.0–11.0	H
Platelet count (PLT)	408	×10^9^/L	150–400	H
Red cell count (RBC)	3.87	×10^12^/L	3.9–5.6	L
Haematocrit (HCT)	0.327	L/L	0.36–0.47	L
Mean cell volume (MCV)	84.4	fL	78–96	
Mean cell haemoglobin (MCH)	27.7	pg	28–34	L
Mean cell haemoglobin concentration (MCHC)	329	g/L	320–360	
White cell differential				
Neutrophils	14.8 (97%)	×10^9^/L	1.9-7.5	H
Lymphocytes	0.3 (2%)	×10^9^/L	1.0-4.0	L
Monocytes	0.2 (1%)	×10^9^/L	0.2-1.0	
Eosinophils	0.0 (0%)	×10^9^/L	0.0-0.4	
Basophils	0.0 (0%)	×10^9^/L	0.0-0.1	

**Table 2:** Renal and liver function tests on admission. GFR: glomerular filtration rate; CKD-EPI: Chronic Kidney Disease Epidemiology Collaboration; ALT: alanine aminotransferase; L: low; H: high.

Test	Result	Unit	Reference range	Flag
Renal function				
Sodium	131	mmol/L	133-146	L
Potassium	4.1	mmol/L	3.5-5.3	
Creatinine	169	µmol/L	49-90	H
GFR (CKD-EPI)	25	mL/min	-	
Liver function				
Total protein	52	g/L	60-80	L
Albumin	24	g/L	35-50	L
ALT	275	IU/L	0-35	H
Alkaline phosphatase	173	IU/L	30-130	H
Total bilirubin	25	µmol/L	5-21	H
Globulin	28	g/L	18-36	

**Table 3:** Inflammatory markers on admission.

Test	Result	Unit	Reference range	Flag
C-reactive protein (CRP)	267	mg/L	0–5	H
Lactate (venous)	2.2	mmol/L	0.5–1.6	H

Four days into admission, she developed swelling of the right leg. D-dimer was raised at 4,894 ng/mL, but Doppler ultrasound excluded a deep vein thrombosis. The following day brought right hip pain and a reduced range of movement. MRI showed a small peri-articular collection with gas foci and surrounding myositis (Figure 1). Subsequent CT extended these findings: multiple gas locules in the right obturator internus muscle extending into the lateral pelvis and an enlarged piriformis muscle (Figure 2), alongside a polypoidal lesion arising from the caecum, an appearance suspicious for an underlying tumour (Figure 3).

**Figure 1:**
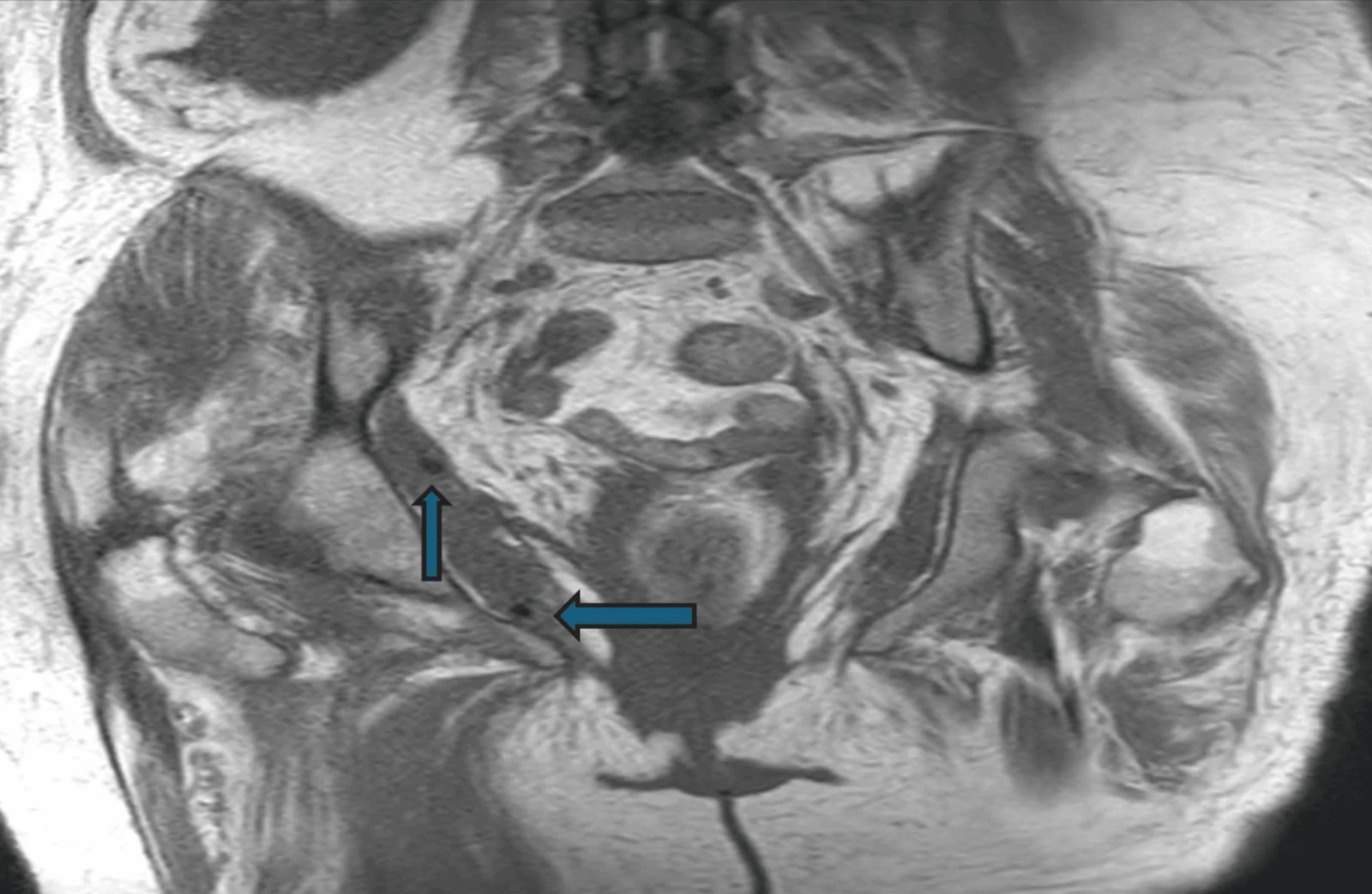
Coronal MRI of the pelvis showing foci of gas within the pelvic soft tissue (arrows).

**Figure 2:**
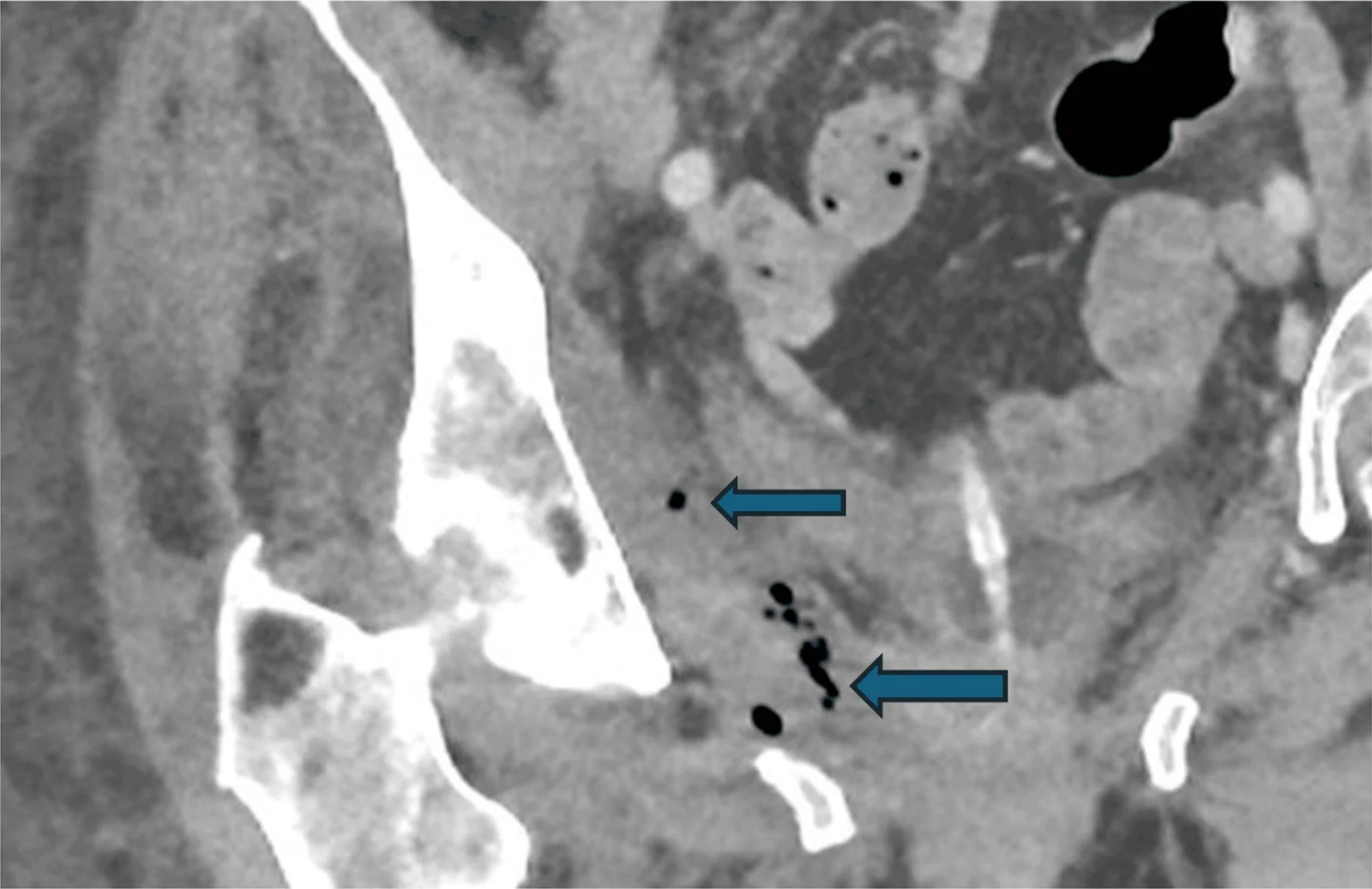
Coronal CT of the pelvis showing multiple gas locules within the right obturator internus muscle extending into the lateral pelvis (arrows).

**Figure 3:**
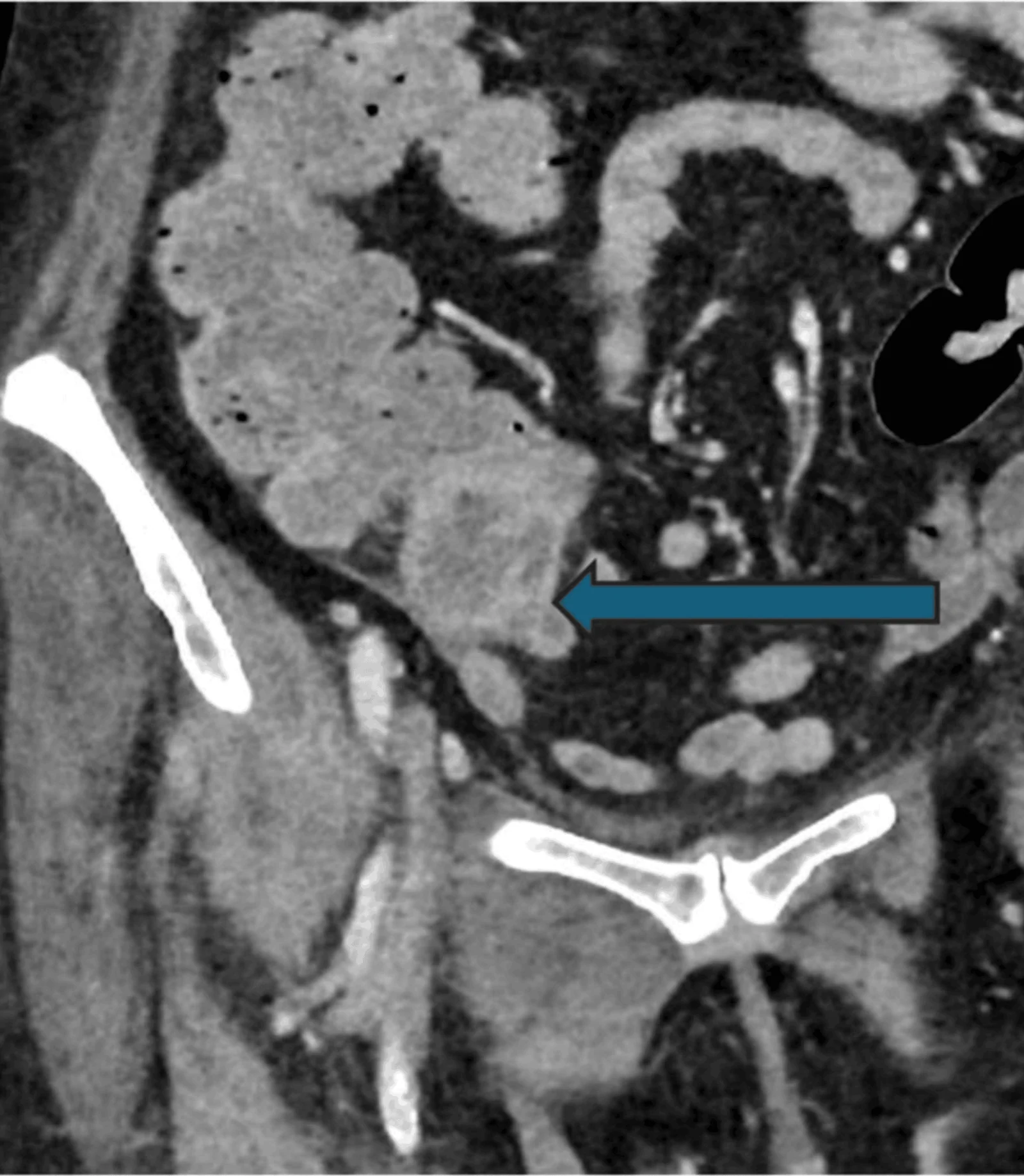
Coronal CT of the abdomen and pelvis showing a polypoidal lesion arising from the caecum (arrow), radiologically staged as T2N0M0 (not histologically confirmed).

Following joint review by the trauma and orthopaedic and interventional radiology teams, the collection was considered too small to drain, and she was managed conservatively with antibiotics. Intravenous meropenem was rationalised to intravenous amoxicillin and metronidazole and subsequently switched to oral co-trimoxazole to complete a total of six weeks of antibiotic therapy. Repeat blood cultures were not taken. However, serial inflammatory markers showed a sustained response to treatment. CRP fell progressively from 267 mg/L on admission to 130, 88, 77, and finally 35 mg/L, and the white cell count fell from 15.3 to 12.4 and then to 7.9 × 10⁹/L, within the reference range. This biochemical improvement, together with her clinical course, supported continued conservative management of the collection without drainage. The caecal appearances were reviewed at the lower gastrointestinal multidisciplinary team meeting, where the consensus was that this looked like adenocarcinoma on imaging. Although the lesion appeared early-stage on imaging, her frailty and burden of comorbidity meant she would not have been a candidate for surgical resection or other cancer-directed treatment. The decision was therefore made not to pursue colonoscopy or biopsy, and she was managed with a palliative approach focused on comfort and quality of life. She was discharged to a nursing home with community palliative follow-up.

## Discussion

*C. septicum* causes disease through a set of potent exotoxins, alpha-toxin chief among them, which break down tissue and can precipitate septic shock and multiorgan failure within hours [3]. What makes it unusual among the clostridia is that it does not require a wound or a surgical field to become established. *C. perfringens* gas gangrene is almost always traumatic in origin; *C. septicum* is not. It crosses directly from a compromised bowel wall into the bloodstream and from there into muscle, joints, or sites further afield, without any obvious point of entry [3-5]. This is essentially what occurred in our patient: no injury, no instrumentation, only gas tracking through the obturator internus and piriformis muscles with a caecal lesion sitting behind it. Given the proximity of the caecum to the right pelvic side wall, direct contiguous spread from the caecal lesion is also possible, and the two routes cannot be reliably distinguished on imaging alone.

The association between *C. septicum* bacteraemia and colorectal malignancy is well established, though it remains underappreciated in day-to-day practice. A large comparative series confirms that caecal and other colonic adenocarcinomas dominate the reported cases [5]. This association has previously come to light through varied routes: endophthalmitis [2], cerebral septic emboli [1], pneumocephalus [6], and distant gas gangrene following biopsy of a caecal mass [3]. Spontaneous myonecrosis is itself a recognised complication of *C. septicum*. What is less often described is involvement of the deep pelvic muscles, as in our patient, where gas in the obturator internus and piriformis presented as leg swelling and hip pain. This prompted the imaging that revealed a suspected occult caecal tumour.

The main diagnostic challenge lay not in the microbiology but in the non-specific initial presentation. Vomiting, diarrhoea, and fever are usually attributed to gastroenteritis or a chest source long before clostridial bacteraemia is considered. Even after* C. septicum* is isolated, clinicians must take a further deliberate step and recognise that the organism points towards the bowel, not just the presenting infection. This step is easily missed unless it is actively sought. In our patient, the right leg swelling and subsequent hip pain led to cross-sectional imaging, which showed both the gas-forming infection and the caecal lesion.

Whether to pursue histological confirmation was a separate consideration. Radiological staging of the caecal lesion as T2N0M0 remains provisional without histology, and in a fitter patient, an early-stage lesion of this kind might have been amenable to curative treatment. Relying on imaging alone carries real limitations. CT is relatively poor at judging depth of bowel wall invasion, particularly in distinguishing T2 from T3 disease, and nodal assessment depends largely on node size, so small-volume nodal involvement can be missed. A polypoidal caecal lesion also has a differential diagnosis beyond adenocarcinoma, including a large benign adenoma, lymphoma, neuroendocrine tumour, and appendiceal pathology. Inflammatory change related to the infection itself may have further altered the appearance of the bowel wall. The diagnosis of malignancy in this patient should therefore be regarded as presumed rather than proven. However, her frailty score, severely impaired cardiac function, and limited physiological reserve meant that she would not have been a candidate for resection, and a tissue diagnosis would not have altered her management [9]. The multidisciplinary team therefore accepted the imaging findings and proceeded directly to palliative planning. This judgement, knowing when further investigation would no longer benefit the patient, was arguably the more difficult decision in this case.

## Conclusions

Bacteraemia with* Clostridium septicum* is rare but carries a high risk of rapidly progressive soft tissue infection and is strongly associated with occult colorectal malignancy. Clinicians who isolate this organism from blood cultures should maintain a low threshold for cross-sectional imaging of both the musculoskeletal system and the colon, even in frail patients in whom curative treatment may ultimately not be feasible, so that the diagnosis, prognosis, and goals of care can be established as early as possible.
